# Surface Plasmon Resonance kinetic analysis of the interaction between G-quadruplex nucleic acids and an anti-G-quadruplex monoclonal antibody

**DOI:** 10.1016/j.bbagen.2018.03.002

**Published:** 2018-06

**Authors:** Sara Lago, Matteo Nadai, Monica Rossetto, Sara N. Richter

**Affiliations:** Department of Molecular Medicine, University of Padua, via A. Gabelli 63, 35121 Padua, Italy

**Keywords:** SPR, Surface Plasmon Resonance, G4, G-quadruplex, Ab, Antibody, RT, Room Temperature, RU, Resonance Units, RII, Refractive Index Increment, Surface Plasmon Resonance, G-quadruplex, Anti-G4 antibody, Specificity, Immobilization, Oriented capturing

## Abstract

**Background:**

G-quadruplexes (G4s) are nucleic acids secondary structures formed in guanine-rich sequences. Anti-G4 antibodies represent a tool for the direct investigation of G4s in cells. Surface Plasmon Resonance (SPR) is a highly sensitive technology, suitable for assessing the affinity between biomolecules. We here aimed at improving the orientation of an anti-G4 antibody on the SPR sensor chip to optimize detection of binding antigens.

**Methods:**

SPR was employed to characterize the anti-G4 antibody interaction with G4 and non-G4 oligonucleotides. Dextran-functionalized sensor chips were used both in covalent coupling and capturing procedures.

**Results:**

The use of two leading molecule for orienting the antibody of interest allowed to improve its activity from completely non-functional to 65% active. The specificity of the anti-G4 antobody for G4 structures could thus be assessed with high sensitivity and reliability.

**Conclusions:**

Optimization of the immobilization protocol for SPR biosensing, allowed us to determine the anti-G4 antibody affinity and specificity for G4 antigens with higher sensitivity with respect to other *in vitro* assays such as ELISA. Anti-G4 antibody specificity is a fundamental assumption for the future utilization of this kind of antibodies for monitoring G4s directly in cells.

**General significance:**

The heterogeneous orientation of amine-coupling immobilized ligands is a general problem that often leads to partial or complete inactivation of the molecules. Here we describe a new strategy for improving ligand orientation: driving it from two sides. This principle can be virtually applied to every molecule that loses its activity or is poorly immobilized after standard coupling to the SPR chip surface.

## Highlights

• Surface Plasmon Resonance (SPR) was used for antibody/G-quadruplex oligos affinity.
• The antibody was efficiently immobilized on SPR chip with two orienting molecules.
• Correct orientation and maintenance of the antibody active site was obtained.
• The key strategy was the use of the antibody/antigen complex during immobilization.
• This general strategy can be applied to every molecule with poor SPR immobilization.

## Graphical abstract

**Figure:**
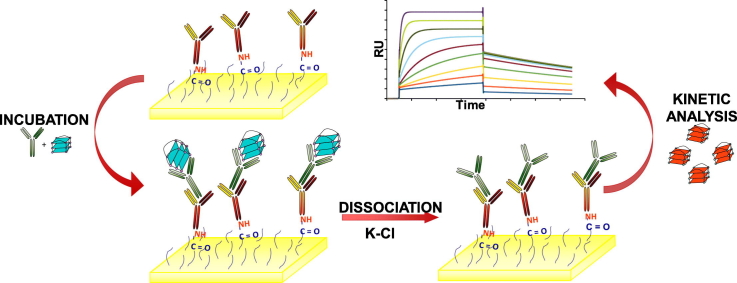

## 1 Introduction

G-quadruplexes (G4s) are non-canonical nucleic acids secondary structures [[1], [2], [3]] that form in guanine (G)-rich DNA and RNA sequences when Gs arrange into stacked planar quartets coordinated by Hoogsteen-type hydrogen bonds and stabilized by metal cations such as K^+^ and Na^+^ [4]. In the human genome, G4s have been reported to play key regulatory [5,6] and pathological roles [7,8]. G4 formation has been reported also in other eukaryotes [9], prokaryotes [10,11] and viruses [[12], [13], [14]]. Thanks to the development and engineering of anti-G4 antibodies, strong evidence of the existence of G4s in cells has been obtained [1,15]. Given the high polymorphism of G4 structures in terms of strand stoichiometry, orientation and topology, and loop length, sequence and position [16,17] the possibility to confirm G4 folding by investigating and characterizing G4 binding to anti G4-antibody is a key benefit. To this aim, Surface Plasmon Resonance (SPR) is a powerful technology: it combines cutting-edge microfluidic and optic implementations to measure biomolecules binding and kinetics [18]. A probe biomolecule is coupled to a functionalized metal surface and a target molecule is flowed into the system. Affinity interactions between the probe and target cause variation in the surface refractive index and thus modify the angle of the incident light, which generates surface plasmon [19]. This shift is tracked, converted to Resonance Units (RU) and shown as binding curve or sensorgram [18]. Several advantages are offered by SPR biosensing: data are acquired in real-time, there is no need for sample labeling and the extremely high sensitivity allows to minimize sample volumes [20]. The SPR technology is widely exploited to characterize antibody-antigens interaction [21,22]. Probe linking to the working surface can be performed according to various strategies, the most common of which is covalent coupling via primary amines [23]. Despite the wide applicability of this chemistry, immobilization by amine coupling is often a limiting step due to generation of heterogeneous surfaces or molecule inactivation [[24], [25], [26]]. Therefore, alternative strategies for oriented immobilization have been devised. A common approach to solve this issue is the exploitation of a bridging molecule that favors correct ligand immobilization. Some of these strategies require ligand conjugation with DNA baits [27] or affinity tags [[28], [29], [30]]. However, the ligand-DNA conjugation process is normally performed through cross-linking reactions that can significantly affect ligand functionality. On the other hand, protein tags introduce a non-negligible steric hindrance possibly modifying the integrity and activity of the tagged molecule.

In the specific case of antibodies, site-directed immobilization has been previously performed through protein A, protein G, secondary antibody recognizing the Fc region or calix[4]arene derivative self-assembled monolayers (SAMs) [[29], [30], [31], [32], [33]]. All these approaches favor an ordered orientation of the antibody of interest and the antigen binding site availability. Further improvements, such as modification of protein G with cysteine residues, allowing the homogeneous orientation of the protein and antibody have been also reported [34,35]. When compared to protein A/G-, secondary Ab-mediated immobilization has been shown to possess improved antigen binding efficiency [31]. We here present an original variation of secondary Ab-mediated immobilization, the novelty of which consists in the occupation of the antigen binding site of the ligand with its antigen. Ideally, the steric hindrance of the antigen protects the antigen binding site from non-specific interactions with the secondary Ab, other ligand molecules or the chip surface. Moreover, once the antigen has been dissociated, it leaves well-distanced Ab molecules facilitating the interaction with newly injected analytes.

The anti-G4 monoclonal antibody 1H6 was employed as ligand Ab [1]. The murine monoclonal antibody was chosen over the phage display-based single chain antibody (BG4) [15] since its constant region can be recognized by species-specific antibodies and it allows the method to be extended to other molecular partners and antibodies.

The new developed immobilization strategy was here shown to highly improve G4/antibody binding characterization.

## 2 Materials and methods

### 2.1 Materials and instrumentation

SPR biosensor analysis was conducted on a Biacore T100 platform with CM5 Series S sensor chip (GE Healthcare, Life Science, Milan, Italy). Amine Coupling kit and Mouse Antibody Capture kit (GE Healthcare, Life Science, Uppsala, Sweden) were respectively exploited for standard amine coupling and the two capturing mediated immobilization strategies. The used oligonucleotides were purchased from Sigma-Aldrich (Milan, Italy) (Table S1). To allow the correct folding of secondary structures, each oligonucleotide was diluted to 20 μM in HEPES-KCl buffer (HEPES 10 mM pH 7.4, KCl 200 mM, EDTA 3 mM), denatured 5 min at 95 °C and then slowly cooled to RT. The mouse monoclonal 1H6 antibody (MW ~155 kDa) was kindly provided by P. M. Lansdorp [1].

### 2.2 Standard amine coupling covalent immobilization

Covalent amine coupling immobilization of 1H6 was performed according to the manufacturer use. Briefly, the flow cell was activated by injection of 1-ethyl-3-(3-dimethylaminopropyl)-carbodiimide (EDC) and N-hydroxysuccinimide (NHS) mixture. After pH-scouting, 1H6 was diluted to 15 ng/μl in NaOAc pH 5.0 and coupled to the surface via exposed primary amines to reach an immobilization level ranging between 1000 and 1200 RU. Finally, ethanolamine was injected to block the unreacted ester groups. A control flow cell was activated and blocked without ligand injection to allow reference subtraction. HEPES-NaCl (HEPES 10 mM pH 7.4, NaCl 150 mM, EDTA 3 mM) was used as immobilization buffer.

### 2.3 Mouse Antibody Capture kit mediated immobilization

Immobilization of the anti-mouse IgG (GE Healthcare, Life Science, Uppsala, Sweden) was performed by amine coupling in HEPES-NaCl buffer at a flow rate of 5 μl/min both on the reference and active flow cell. The EDC/NHS mixture was injected for 420 s to activate the chip surface. The anti-mouse IgG diluted to 40 ng/μl in HEPES-NaCl buffer was injected for 600 s and the unreacted ester groups were then blocked by 420 s injection of ethanolamine. Finally, the unbound ligand was washed away through three consecutive injections of 10 mM glycine, pH 1.7 (60 s, 50 μl/min). Immobilization levels between 3000 and 6000 RU were typically obtained and then primed with HEPES-KCl buffer. 1H6 was diluted to 15 ng/μl in the same buffer and captured by 600 s injection at a flow rate of 5 μl/min on the active anti-mouse IgG immobilized flow cell. Finally, 1 M KCl was injected (20 s, 30 μl/min flow rate) to dissociate the non-specifically bound ligand. Typical capturing levels ranged between 800 and 1200 RU.

### 2.4 G-quadruplex complex-mediated immobilization

The anti-mouse IgG was immobilized on reference and active flow cells as described in the section above. After 1 h incubation at RT in the presence of 10 folds of G4 folded oligonucleotide (*Oxy2*), 1H6 was captured on the active flow cell. The final injection of 1 M KCl (20 s, 30 μl/min flow rate) was used to dissociate *Oxy2* from the captured 1H6. Typical capturing levels ranged between 800 and 1200 RU.

### 2.5 Kinetic analysis and data evaluation

Each analyte was tested in the concentration range 0.250–8 μM. For *Oxy2*, we tested two additional low concentrations (0.125 and 0.062 μM) to cover the whole kinetic points. Samples were injected for 340 s at a flow rate of 20 μl/min and dissociated for 340 s. HEPES-KCl buffer was used as running buffer and for samples dilution. Surface regeneration at the end of each analyte concentration was performed by 20 s injection of 1 M KCl at a flow rate of 30 μl/min. Complete regeneration of 1H6 for successive immobilizations was instead performed with 180 s injection of 10 mM glycine pH 1.7 (GE Healthcare, Uppsala, Sweden). BiaEvaluation Software was used for data analysis. The likelihood of fittings was assessed through the statistical parameters of Chi^2^ and U-value [36,37]. The percentage of active surface was measured as a proportion of either the maximum observed response (*R*_*max*_*OBS*), or the kinetic fitting-determined maximum response (*R*_*max*_*FIT*), to the theoretically expected maximum response (*R*_*max*_*EXP)*, where*:*

(1) $$R_{\max}\mathrm{EXP}=\left({\mathrm{MW}}_{A}/{\mathrm{MW}}_{L}\right)\times {\left(\mathrm{\delta n}/\delta_{C}\right)}_{A}/{\left(\mathrm{\delta n}/\delta_{C}\right)}_{L}\times {\mathrm{RU}}_{I}\times S$$

*R*_*max*_*EXP* is the expected maximal response of the interacting molecules expressed in RU; *MW*_*A*_ and *MW*_*L*_ are the molecular weights of the analyte and ligand, respectively; *RU*_*I*_ is the amount of immobilized ligand expressed in RU; *S* is the stoichiometry of the interaction; *(δn/δ*_*C*_*)*_*A*_ and *(δn/δ*_*C*_*)*_*L*_ are the refractive index increments (RII) for the analyte and ligand, respectively. RII is an optical property of the molecule that depends on the refractive index at the surface (*n*) and the concentration (*C*) of the binding partners. For protein-nucleic acids interactions, RII ratio can be approximated to 1 and discrepancies from the expected *R*_*max*_ can be ascribed to the surface activity of the immobilized SPR chip surface [38]. Often, differences between the composition of the analyte stock buffer and the SPR running buffer used for diluting the injected analytes generate the so called bulk effect [37,39], which appears as a sharp jump of the RU response at the very beginning and after the end of the injection cycle. To obtain an appropriate fitting of experimental curves, a standard value of 1/5 of the experimental responses is normally used to correct experimental data where a bulk effect is detected [40]. The actual refractive index correction detected by the sensorgrams fitting is reported in Table S3. Moreover, to obviate this problem, *R*_*max*_*OBS* has been measured 7 s after the injection stop, approximating the analyte binding level at a kinetic point not affected by bulk shifts [41,42].

Kinetic constants were obtained from 1:1 L fitting (*un3* and *bcl-*2) or heterogeneous analyte fitting (*Oxy2*) of sensorgrams, applying default bulk correction, after double subtraction of buffer and reference flow cell responses. Each immobilization strategy and kinetic analysis was repeated at least three times. Baseline stability and influences of small baseline deviations on the goodness of fitting have been measured as:

(2-3) $$R\varepsilon i/R_{\mathrm{BL}}\times 100\;\text{where}\;R\varepsilon i=|\mathrm{Ri}-R_{\mathrm{BL}}|$$

*R*_*BL*_ is the measured baseline before injection of the analyte and *Ri* is the baseline measurement after each regeneration.

### 2.6 Electrophoretic mobility shift assay (EMSA)

Oligonucleotides were 5′-end labeled with [γ-^32^P]ATP by T4 polynucleotide kinase after 30 min incubation at 37 °C, purified using MicroSpin G-25 columns (GE Healthcare Europe, Milan, Italy), resuspended in lithium cacodylate buffer 10 mM pH 7.4 with KCl 100 mM, heat denatured and folded. Samples were run in 20% non-denaturing polyacrylamide gels for ~ 24 h at 60 V. Gels were fresh impressed and visualized by phosphorimaging (Typhoon FLA 9000, GE Healthcare Europe, Milan, Italy). Quantification of shift bands was performed by ImageQuant TL Software (GE Healthcare Europe, Milan, Italy).

### 2.7 Circular dichroism spectroscopy (CD)

Oligonucleotides were diluted to a final concentration of 4 μM in lithium cacodylate buffer (10 mM, pH 7.4) and KCl 100 mM. After heat denaturation samples were folded at room temperature overnight. CD spectra were recorded on a Chirascan-Plus (applied Photophysics, Leatherhead, UK) equipped with a Peltier temperature controller using a quartz cell of 5 mm optical path length. The spectra were recorded over a wavelength range of 230–320 nm at a temperature of 20 °C. Acquired spectra were baseline corrected for signal contribution due to the buffer and the observed ellipticities converted to mean residue ellipticity (θ) = deg. × cm2 × dmol−1 (mol ellip).

## 3 Results and discussion

We here aimed at measuring by SPR biosensing the kinetic and thermodynamic binding affinities of a series of G4s to the anti-G4 monoclonal antibody 1H6 [1]. We tested three G4 oligonucleotides found in the ciliate *Oxytricha nova* (*Oxy2*) [43], the herpes simplex virus-1 (HSV-1) (*un3*) [12] and the mammalian cell (*bcl-2*) [44]. *Oxy2* is the G4 oligonucleotide used to produce 1H6 in immunized mice and was here employed as the positive control [1]. Three other oligonucleotides unable to form G4, i.e. a double-stranded (*ds*), a single-stranded (*ss*) and a hairpin DNA (*hp*) were used as negative controls.

**Fig. 1:**
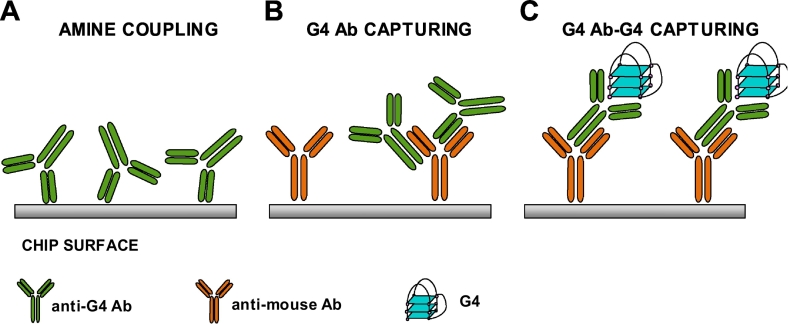
Representation of 1H6 antibody orientation on the chip surface according to the different immobilization strategies. A) Direct amine coupling. B) Anti-mouse antibody mediated capturing. C) Anti-mouse antibody mediated capturing following the incubation of 1H6 with a G4 partner.

Three different methods of ligand (1H6) immobilization on the SPR sensor chip were compared (Fig. 1).

### 3.1 Standard amine coupling of 1H6

Amine coupling chemistry is one of the most commonly used immobilization strategies in SPR biosensing [23]. Ester reactive groups on the sensor chip dextran matrix are activated and the ligand of interest is then flowed on the chip cell to allow primary amines coupling.

**Fig. 2:**
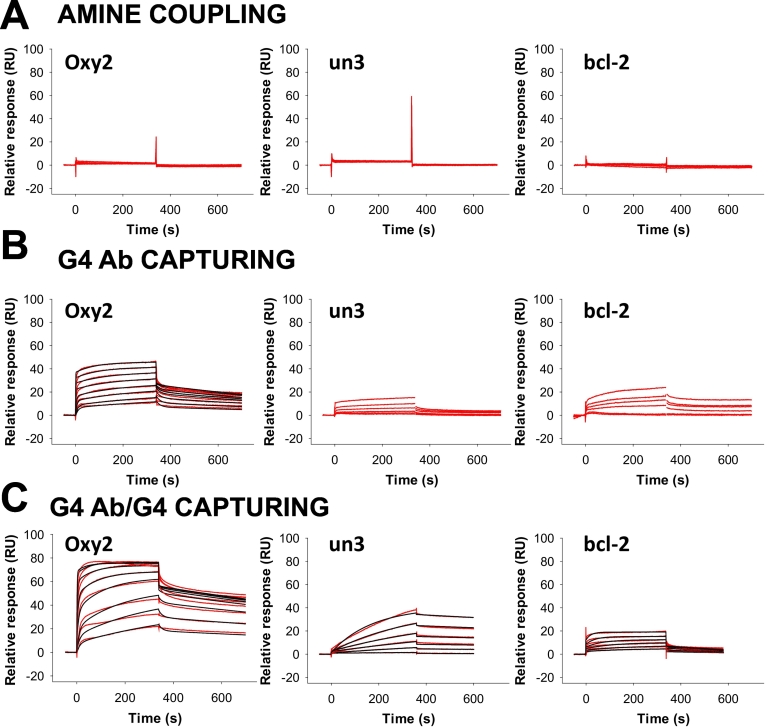
Binding analysis of 1H6-G4 nucleic acids interaction after A) 1H6 direct amine coupling, B) anti-mouse mediated 1H6 capturing, C) anti-mouse mediated capturing of the 1H6-G4 complex. *Oxy2* G4 analyte was tested in the concentration range 62.50 nM–8.00 μM, while for *un3* and *bcl-2* the 250 nM–8.00 μM range was sufficient to cover the kinetic spectrum. Recorded sensorgrams are shown in red, while fitting curves are in black. Fitting curves were not reported when they did not fit or the kinetic constants were outside the sensitivity of the instrument. Parameters for the goodness of fittings are reported in Table S1.

For kinetic analysis, low-density ligand surface is recommended to limit mass transfer effect (i.e. the difference in analyte diffusion speed in the flowing solution and through the chip matrix) [45], but as a compromise, a good signal-to-noise ratio should be maintained [46]. The optimal level of ligand to be immobilized (*RU*_*immobilized*_) can be calculated rearranging Eq. (1). According to these indications, we immobilized about 1000 RU of 1H6 by amine coupling (Fig. S2A). In this condition, G4 binding molecules (analytes) are expected to reach an *R*_*max*_ value ranging between 50 and 130 RU. However, when the G4-forming oligonucleotides *Oxy2*, *un3* and *bcl-2* (and the non-G4 oligonucleotides) were flowed on the immobilized 1H6, no binding was observed (Fig. 2A and S3A).

We reasoned that these negative results likely derived from loss of binding activity of the chip-immobilized 1H6. This could be due to: i) 1H6 denaturation caused by its dilution in low pH buffer; ii) presence of basic residues in the G4 binding site of 1H6 that have reacted with the activated dextran matrix impairing 1H6 ability to bind G4s (Fig. 1A). In support of this hypothesis, portions of the variable heavy and light chains of 1H6 have been reported to contain basic amino acids that directly interact with nucleic acids [1]; iii) reduction of 1H6 “breathing” and steric hindrance that interferes with G4 recognition [47]; iv) a combination of all the above.

### 3.2 Antibody capture-mediated immobilization of 1H6

To avoid the above highlighted problems, we shifted to a capturing immobilization strategy consisting in the covalent immobilization on the chip of an antibody that recognizes the species-specific region of 1H6 (Fig. 1B). We bound an anti-mouse polyclonal antibody (GE Healthcare, Uppsala, Sweden) on the sensor chip through amine coupling (Fig. S2 B). We next injected 1H6, which is captured via its Fc region or exposed constant chains (Fig. S4 A).

**Fig. 3:**
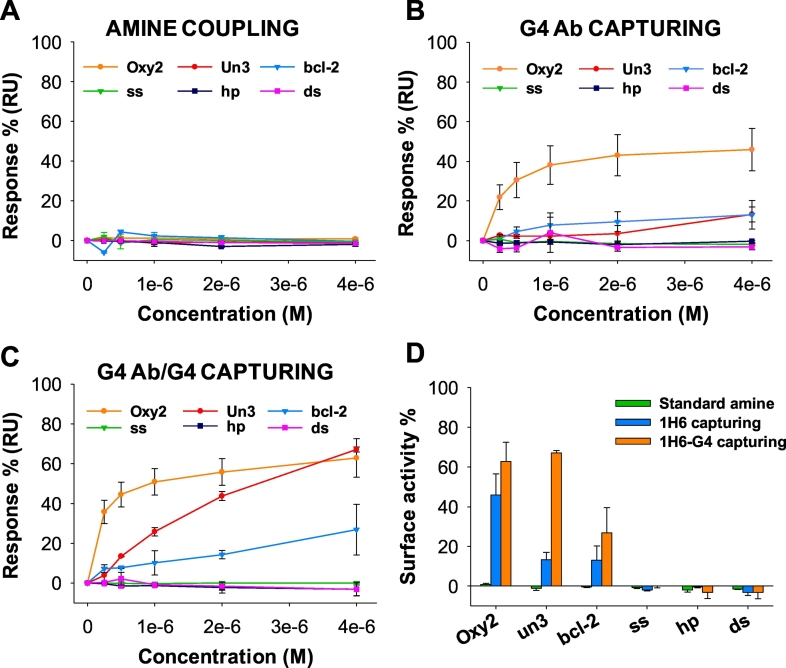
Sensor chip surface activity. 1H6 surface activity was measured as percentage of *R*_*max*_*OBS* to the theoretically expected *R*_*max*_ obtained for each tested analyte (0 nM−4.00 μM) after A) direct amine coupling of 1H6, B) anti-mouse mediated 1H6 capturing and C) anti-mouse mediated capturing of previously formed 1H6-G4 complex. Panel D) shows the comparison of surface activity determined at analyte concentration (4.00 μM) for the three immobilization strategies as indicated.

With respect to covalent amine coupling, the described capturing method has the advantage to work at physiological pH and to be an oriented strategy. In fact, 1H6 is directly diluted in the running buffer, and the pre-immobilized anti-mouse antibody reduces orientation heterogeneity, which is typical of the amine coupling chemistry [26,48]. Another advantage of the capturing methods with respect to covalent coupling is that the flow cell can be regenerated and re-immobilized with the ligand (1H6). We set times and flow rates to obtain the desired level of about 1000 RU immobilized 1H6 (Figs. S2A and S4A). Binding analysis in the same conditions used in the standard amine coupling method was performed on the 1H6-captured flow cell. A detectable binding response was obtained for each of the tested G4s, while no binding was observed for the non-G4 negative controls (Figs. 2B and S3B). However, the registered sensorgrams did not allow reliable determination of thermodynamic and kinetic parameters for *un3* and *bcl-2* due to low responsivity of the immobilized surface (Table 1). Indeed, we found that the best analyte (*Oxy2*) reached only 45% of the theoretically expected *R*_*max*_ at or close to saturating concentration (Fig. 3B). We reasoned that such a reduced binding capacity of the immobilized ligand could be due again to heterogeneous orientation of 1H6 G4 binding site. Being the capturing anti-mouse antibody a polyclonal immunoglobulin, there are several portions of 1H6 Fc region that can be recognized. In addition, differently oriented 1H6 molecules, together with the crowding of the capturing surface, can impair or hide the availability of the G4 binding site (Fig. 1B). Thus, despite the anti-mouse antibody-mediated capturing of 1H6 improved the detection of G4 analytes binding, nonetheless it was not sufficient to obtain robust affinity data.

**Table 1:** Kinetic parameters for 1H6-G4 interaction.

	Amine coupling			G4 Ab capturing			G4 Ab/G4 capturing			
	ka (Ms^−1^)	kd (s^−1^)	KD (nM)	ka (10^4^ Ms.^−^^1^)	kd (10^−4^ s^−1^)	KD (nM)	ka (10^4^ Ms.^−^^1^)	kd (10^−4^ s^−1^)	KD (nM)	
*Oxy2*	nd	nd	nd	29.50 ± 0.92	6.99 ± 1.73	26.13 ± 0.51	5.83 ± 0.40	5.13 ± 1.59	10.78 ± 3.38	tetra
				110.60 ± 56.40	2385.26 ± 1774.46	74.45 ± 3.85	96.30 ± 41.91	438.73 ± 141.40	64.33 ± 15.15	bi
*un3*	nd	nd	nd	nd	nd	nd	0.07 ± 0.01	3.30 ± 0.10	479.65 ± 2.75	
*bcl-2*	nd	nd	nd	nd	nd	nd	0.58 ± 0.01	30.10 ± 11,0	518.05 ± 20.20	

nd = non detectable; uncertainties of the reported values are calculated as the standard deviation of at least two experimental replicates obtained with independent ligand immobilizations.

### 3.3 Antibody capture-mediated immobilization of 1H6-G4 complex

We next developed a doubly oriented capturing strategy for 1H6 immobilization. The anti-mouse polyclonal antibody was covalently immobilized on the sensor chip surface as described above (Figs. 1B and S2C) and used as the primary leader to orient 1H6 immobilization on the chip. In addition, 1H6 was incubated in the presence of its natural antigen *Oxy2* G4 to allow complex formation and have a secondary leader for correct orientation. The 1H6-G4 pair was then injected on the anti-mouse-immobilized surface (Figs. 1C and S4B). The G4 complexed to 1H6 sterically hinders the interaction of the G4 binding site of 1H6 with the anti-mouse antibody; moreover, it weakens surface crowding effects, which would reduce accessibility of 1H6 to its G4 binding partners. An effect of the 1H6-complexed G4 on the interaction with the anti-mouse antibody can also be deduced from the slope of the capturing association curve. Comparing the curves obtained during free 1H6 or G4-1H6 capturing, we observe that the second interaction has a slower association rate (Figs. S4A and S4B). This is likely due to the presence of the G4, which reduces the availability of some 1H6 binding sites recognized by the immobilized anti-mouse antibody. Being the anti-mouse antibody/1H6 interaction much stronger than the 1H6/G4 one, *Oxy2* G4 can be dissociated from 1H6 with the injection of a high ionic strength buffer (KCl 1 M) without perturbation of the system stability so that the analytes of interest can be tested for complete kinetic experiments (*ka* and *kd* measurements). The amount of captured 1H6 in the two capturing strategies was comparable, as shown by the RU after KCl 1 M regeneration in the capturing curves (Fig. S4). However, in the G4-1H6 capturing, a mild drop of about 100 RU was obtained after regeneration (Fig. S4B): this was attributed to the dissociation of *Oxy2* G4, as it corresponded to the theoretical *Oxy2* amount, expressed in RU, necessary to saturate the captured 1H6.

Capturing strategies generally produce less stable binding of the ligand on the chip with respect to covalent coupling. We demonstrated however that the 1H6 baseline (i.e. the absolute RU response measured before injection of each analyte concentration when only buffer flows on the flow cell) remained stable over repeated dissociations events, indicating that our regeneration strategy efficiently removes analytes without affecting the chip-captured ligand. Precisely, variations recorded on the same flow cell had a mean contribution lower than 0.4% on the sensorgram fitting equation [49], therefore allowing reliable determination of kinetic constants over more than 80 injection cycles (Fig. S5).

About 1000 RU of 1H6 were immobilized also with this improved capturing method (Figs. S2C and S4B). Kinetic binding analysis showed enhanced response for each of the G4 oligonucleotide (Fig. 2C), while no binding was recorded for the non-G4 controls (Fig. S3C). Thermodynamic constants evaluated by sensorgrams fitting (Fig. 2C) indicated that 1H6 bound *Oxy2* with the highest affinity (KD_tetra_ 9.16 ± 2.81 nM, KD_bi_ 41.73 ± 13.15 nM), followed by *bcl-2* (375.50 ± 1.46 nM) and *un3* (479.65 ± 2.75 nM) (Table 1).

The binding curves of *Oxy2* G4 displayed a complex behavior, characterized by a slow rise of the association phase and a fast dissociation followed by a slower decrease in the response. This biphasic dissociation can be explained by the presence of two different G4 species formed by the *Oxy2* analyte. Two distinct shift bands are indeed visible in electrophoresis mobility shift assay (EMSA) performed at the same oligonucleotide concentrations used in SPR binding analysis (Fig. S6). The two species were ascribed to the bi- and tetramolecular forms of *Oxy2* G4. The parallel topology recorded by CD spectroscopy for *Oxy2*, folded at a concentration corresponding to the presence of the sole upper band in EMSA native gel, supports the predicted stoichiometry. It has indeed been previously demonstrated that the tetramolecular form of *Oxy2* adopts a parallel conformation, while the dimer folds as antiparallel G4 [[50], [51], [52], [53], [54]]. Notably, the relative abundance of the prevalent species in solution is highly dependent on the oligonucleotide concentration. The effective concentration of the two *Oxy2* species has been determined by quantification of the corresponding EMSA bands (Table S4). To obtain accurate fitting of *Oxy2* interaction with 1H6 antibody, the molecular weight and concentration of the bi- and tetramolecular G4s have to be taken into account. The heterogeneous analyte model has been therefore applied to obtain reliable kinetic constants (Tables 1 and S2). The KD determined for *Oxy2* is below the lowest tested analyte concentration, it should therefore be considered as only representative of the affinity order of magnitude. Both statistical parameters and EMSA experimental results support however the validity of the *Oxy2* kinetic characterization (Fig. S6 and Table S2).

### 3.4 Comparison of surface activity

To quantify the performance of the three different immobilization strategies of 1H6 on SPR sensor chips, we measured the binding level of each tested G4 analyte concentration as percentage of the expected *R*_*max*_*EXP*. For this calculation both the observed (*R*_*max*_*OBS*) and fitting-determined (*R*_*max*_*FIT*) values were considered. Comparable results were obtained with the two parameters, supporting the rigorousness of the data. In line with the experimental curves, only *un3* fitting *R*_*max*_ is substantially higher than the corresponding *R*_*max*_*OBS*. Indeed, at the chosen concentration, *un3* does not reach ligand saturation. For this reason, *R*_*max*_*OBS* is preferable to compare surface activities, being more indicative of the actual values at the chosen concentration (Table S2 and Fig. 2). One G4 molecule was considered binding to one molecule of 1H6 antibody, given that G4 folded oligonucleotides are rather large (cell dimensions of a trigonal crystal of bimolecular *Oxytrichia nova* telomeric G4 are a = 27.54 Å, b = 27.54 Å, c = 145.81 Å) [53] and that the distance between the two antigen-binding sites of IgG antibody is limited (117–134 ± 40 Å) [55]. As discussed above, covalent amine coupling impaired ligand (1H6) functionality (Fig. 3A and D). Simple capturing of 1H6 produced a maximal observed response of about 45% for *Oxy2*, the highest affinity analyte, and of 13% for both *un3* and *bcl-2* (Fig. 3B and D). 1H6 capturing following incubation with a G4 molecule highly improved ligand functionality, especially for the lower affinity analytes: 63% surface functionality was indeed reached for *Oxy2*, 67% for *un3*, and 27% for *bcl-2* (Fig. 3C and D). The inability of the latter strategy to reach 100% of surface functionality can be partially explained by the competition between the G4 and the anti-mouse antibody for the common binding sites on 1H6 during the capturing process. A fraction of the 1H6-G4 complexed molecules can indeed dissociate and free 1H6 “wrongly” interact with the anti-mouse antibody. The hindrance made by the stably 1H6-complexed G4s is however sufficient to greatly increase the desired orientation of 1H6 molecules. The surface functionality is indeed about 20% higher with respect to that obtained after capturing of free 1H6. These data therefore confirm that G4-mediated capturing of 1H6 enhances analyte binding sensitivity and allows reliable evaluation of the binding of low-affinity analytes.

## 4 Discussion

SPR is a highly sensitive, fast and low sample consuming technique for the thermodynamic and kinetic characterization of biomolecules interaction. These features make SPR the preferable choice with respect to other in vitro techniques such as ELISA, which does not allow an absolute measurement of kinetic constants, is less sensitive and more expensive in terms of required samples and time. SPR is therefore the most suitable technology to verify the real specificity and affinity of the anti-G4 antibody 1H6 for different G4 antigens. This issue is a fundamental assumption for the further exploitation of the antibody in *in vivo* experiments for the monitoring of G4 structures in a cellular context.

The SPR technology requires that one of the analyzed molecules is immobilized on a functionalized metal surface. The most commonly used strategy is covalent amine coupling, which virtually works for every molecule with exposed primary amines [23]. However, it often leads to heterogeneous orientation of the ligand molecule and alteration of its activity. Several alternative immobilization or non-covalent capturing strategies can be exploited, such as thiol coupling and molecular linkers directed immobilization [22,24,26,30].

We here tested three immobilization strategies for the immobilization of 1H6 antibody on dextran functionalized sensor chip. Each of them leads to a satisfying level of bound ligand, which resulted however functional only after oriented capturing. Non-covalent capturing of the ligand provides several other advantages: i) it allows to work at physiological pH, therefore preserving the integrity and functionality of the ligand; ii) the pH scouting procedure is not necessary allowing to spare time and sample; iii) the captured ligand can be completely regenerated and re-immobilized, so the same flow cell can be used several times; iv) lower ligand volume is required to obtain the same immobilized level.

## 5 Conclusion

The newly developed doubly oriented method we described here allows the accurate and sensitive characterization of binding between the anti-G4 antibody 1H6 and different G4 antigens. The SPR immobilization of a ligand mediated by previous incubation with an analyte (Fig. 1C) can be applied to proteins in general and to molecules that require correct folding. It can also be applied to position on the chip surface every type of molecules so that the binding site is correctly exposed and available for analyte binding.

## Transparency document

Transparency document Image 1

## Appendix A Supplementary data

Supplementary material Image 2
