# Supplementary material for: Surface Plasmon Resonance kinetic analysis of the interaction between G-quadruplex nucleic acids and an anti-G-quadruplex monoclonal antibody
Source: Biochim Biophys Acta. 2018 Jun;1862(6):1276–82. doi: 10.1016/j.bbagen.2018.03.002 (PMC5988565; doi:10.1016/j.bbagen.2018.03.002)
Supplement: Supplementary file 2 — Supplementary material [file mmc2.docx]

**A new protocol for kinetic analysis of 1H6 monoclonal antibody and G-quadruplex nucleic acids using Surface Plasmon Resonance**

Sara Lago, Matteo Nadai, Monica Rossetto and Sara N. Richter*

Department of Molecular Medicine, University of Padua, via Gabelli 63, 35121 Padua, Italy.

Table reporting the nucleotidic sequence, molecular weight of the monomeric species (MW) and type of secondary structure adopted by all oligonucleotides used in the present study. Additional figures illustrating pH-scouting and immobilization/capturing sensorgrams, binding analysis sensorgrams for all negative control oligonucleotides and baseline stability for the double-capturing method described in the paper. EMSAand CD experiments for the assessment of *Oxy2* G4 species and folding stoichiometry.

| **Name** | **Sequence 5'-3'** | **MW (Da)** | **Structure** |
| --- | --- | --- | --- |
| *Oxy2* | TTTTGGGGTTTTGGGG | 5005 | Bi-Tetramolecular G4 [1-3] |
| *Un3* | GGGAGGAGCGGGGGGAGGAGCGGG | 7695 | Intramolecular G4 [4] |
| *bcl-2* | AGGGGCGGGCGCGGGAGGAAGGGGGCGGGAGCGGGGCTG | 12436 | Intramolecular G4 [5] |
| *ss* | AAAAACTACTGCACGCTCGCTACGACGACACTGTCGCGCATACAAGCTGCAAAAA | 16850 | Single strand helix |
| *hp* | CGCAGCGTGGCTTTGTTTGCCACGCTGCG | 8896 | Hairpin |
| *ds* | GGATGTG AGTGTGAGTGTGAGG + complementary sequence | 6504+6967 | Double strand helix |

**Table S1. Oligonucleotides used in the new protocol.** The name, sequence, molecular weight and reported secondary structure along with references of all oligonucleotides are reported

**Figure S1. pH scouting procedure applied before direct amine coupling of 1H6.** Ligand pre-concentrations at various acid pH were injected on the non-activated chip surface to evaluate the electrostatic attraction to the surface and choose the more suitable èH for successful coupling reaction. To this aim, 1H6 was diluted to 20 ng/μl in sodium acetate (NaOAc) pH 4.0, 4.5, 5.0 and 5.5, and injected for 180 sec at a flow rate of 5 μl/min.

**1H6**

**EDC/NHS**

**Pre-conc**

**Ethanolamine**

**A**

**1H6 AMINE COUPLING**

**B**

**ANTI-MOUSE AMINE COUPLING**

**C**

**ANTI-MOUSE AMINE COUPLING**

**Figure S2. Covalent amine coupling procedure.** Immobilization sensorgrams obtained for **A)** 1H6 direct amine coupling, **B)** and **C)** anti-mouse antibody covalent amine coupling for subsequent 1H6 or 1H6-G4 capturing respectively.

**ss**

**hp**

**ds**

**B**

**1H6 CAPTURING**

**C**

**1H6-G4 CAPTURING**

**ss**

**hp**

**ds**

**A**

**AMINE COUPLING**

**ss**

**hp**

**ds**

**Figure S3. Sensorgrams obtained for kinetic analysis of 1H6 interaction with non-G4 nucleic acids. A)** Sensorgrams obtained after 1H6 direct amine coupling. **B)** Sensorgrams obtained after anti-mouse mediated 1H6 capturing. **C)** Sensorgrams obtained after anti-mouse mediated capturing of the previously formed 1H6-G4 complex.

**1H6-G4 capturing**

**Regeneration**

**B**

**1H6-G4 CAPTURING**

**A**

**1H6 capturing**

**Regeneration**

**1H6 CAPTURING**

**Figure S4. Capturing procedure. A)** Free 1H6 or **B)** 1H6-G4 complex was captured by the anti-mouse antibody previously immobilized on the sensor chip surface.

|  | **G4 Ab CAPTURING** | | | **G4 Ab/G4 CAPTURING** | | |
| --- | --- | --- | --- | --- | --- | --- |
|  | ***Oxy2*** | ***un3*** | ***bcl-2*** | ***Oxy2*** | ***un3*** | ***bcl-2*** |
| **R_max_ EXP (RU)** | 119.22 ± 11.87 | \ | \ | 130.47 ± 11.76 | 40.74 ± 4.89 | 73.38 ± 8.24 |
| **R_max_ OBS (RU)** | 56.23 ± 15.15 | \ | \ | 80.6 ± 8.00 | 27.30 ± 2.80 | 20.75 ± 11.55 |
| **R_max_ FIT (RU)** | 60.12 ± 15.59 | \ | \ | 83.04 ± 9.38 | 50.48 ± 8.74 | 15.68 ± 8.12 |
| **Surface activity OBS (%)** | 45.69 ± 7.45 | \ | \ | 62.84 ± 11.80 | 67.16 ± 1.19 | 26.85 ± 12.73 |
| **Surface activity FIT (%)** | 47.80 ± 8.95 | \ | \ | 64.04 ± 8.96 | 123.12 ± 6.67 | 20.38 ± 8.78 |
| **Chi^2^** | 4.11 ± 2.61 | \ | \ | 4.39 ± 12.55 | 0.49 ± 0.02 | 0.52 ± 0.18 |
| **U-value** | \ | \ | \ | \ | 5.00 ± 1.00 | 8.00 ± 4.00 |

**Table S2. Values of expected and observed R_max_, surface activity and fitting likelihood parameters.** *Surface activity OBS* and *Surface activity FIT* were calculated as a proportion between the expected and, respectively, the observed and fitting-determined R_max_ values. For *Oxy2* G4 the R_max_EXP was calculated using the molecular weight of the tetramolecular form, since at the saturating concentrations it is the prevalent topology **(see Figure S6 and Table S3)**. Chi^2^ and U-value are representative of the goodness of fittings [6, 7].

|  | **G4 Ab CAPTURING RI (RU)** | | | **G4 Ab/G4 CAPTURING RI (RU)** | | |
| --- | --- | --- | --- | --- | --- | --- |
| **Concentration (nM)** | ***Oxy2*** | ***un3*** | ***bcl-2*** | ***Oxy2*** | ***un3*** | ***bcl-2*** |
| 62.50 | -1.41 ± 0.38 | \ | \ | -0.28 ± 0.21 | \ | \ |
| 125 | -0.32 ± 1.34 | \ | \ | 2.89 ± 2.00 | \ | \ |
| 250 | 0.79 ± 0.61 | \ | \ | 2.33 ± 1.18 | 0.87 ± 0.00 | 1.83 ± 0.63 |
| 500 | 4.61 ± 3.13 | \ | \ | 4.44 ± 3.03 | 1.70 ± 0.44 | 2.44 ± 0.96 |
| 1000 | 9.50 ± 2.69 | \ | \ | 10.29 ± 2.56 | 3.24 ± 0.87 | 3.43 ± 1.37 |
| 2000 | 14.31 ± 2.95 | \ | \ | 15.70 ± 2.70 | 4.69 ± 1.96 | 4.63 ± 1.60 |
| 4000 | 17.87 ± 2.93 | \ | \ | 19.10 ± 2.34 | 5.56 ± 3.72 | 6.46 ± 2.01 |
| 8000 | 21.03 ± 1.62 | \ | \ | 20.43 ± 1.66 | 6.76 ± 5.45 | 3.88 ± 2.74 |

**Table S3. RI values used for bulk correction in sensorgrams fitting.** The reported RI values were obtained from the fitting of experimental curves through a heterogeneous model for *Oxy2* and 1:1 binding for *un3* and *bcl-2*. The determined RI represents the bulk contribution of the oligonucleotides stock buffer at the different analyte concentrations used in the kinetic analysis. The reported values are the average of at least two replicates. Some of the lowest *Oxy2* concentration RI values are negative, but are zero or close to zero when considering the experimental error, suggesting the absence of bulk contribution for the most diluted analytes.

**Figure S5. Effect of baseline drift on sensorgram fitting equation.** Contribution of baseline drift on the determination of kinetic constants through 1:1 Langmuir binding model calculated for more than 90 analyte injection cycles on the same flow cell immobilized through 1H6-G4 complex capturing.

**Figure S6. EMSA experiment of *Oxy2*.** The *Oxy2* G4 was folded at the same set of concentrations used in SPR binding analysis in the range 15 nM-16µM. Two different species, ascribed to the bi- and tetramolecular G4 appear to form in a concentration dependent manner. The first three lanes where loaded with molecular weight markers.

| **% abundance** | | **Concentration (nM)** | | |
| --- | --- | --- | --- | --- |
| ***tetra*** | ***bi*** | ***tetra*** | ***bi*** | **Total** |
|  | 100 |  | 15.12 | 15.12 |
| 11.22 | 88.78 | 3.51 | 27.74 | 31.25 |
| 59.52 | 40.48 | 37.20 | 25.30 | 62.50 |
| 64.89 | 35.11 | 81.11 | 43.89 | 125 |
| 71.54 | 28.46 | 178.85 | 71.15 | 250 |
| 76.55 | 23.45 | 382.75 | 117.25 | 500 |
| 77.85 | 22.15 | 778.50 | 221.50 | 1000 |
| 80.74 | 19.26 | 1614.80 | 385.20 | 2000 |
| 86.28 | 13.72 | 3451.20 | 548.80 | 4000 |
| 89.16 | 10.84 | 7132.80 | 867.20 | 8000 |
| 92.74 | 7.26 | 14838.40 | 1161.60 | 16000 |

**Table S4. Percentage abundance and estimated concentration of the bi- and tetramolecular G4 species formed by *Oxy2* in the presence of 100 mM K^+^.** The reported values were obtained from the quantification of shift bands obtained by native EMSA experiment **(Figure S6)**.

**Figure S7. Circular Dichroism spectra of *Oxy2.*** The spectrum was recorded at 20 °C after folding *Oxy2* (4 μM) overnight in the presence of 100 mM KCl. The displayed positive peak at 265 nm and a negative peak at 240 nm are typical of a G4 parallel topology, which has been reported for the tetramolecular species [1, 2].

**REFERENCES**

[1] T. Miura, J.M. Benevides, G.J. Thomas, Jr., A phase diagram for sodium and potassium ion control of polymorphism in telomeric DNA, Journal of molecular biology, 248 (1995) 233-238.

[2] L. Oganesian, I.K. Moon, T.M. Bryan, M.B. Jarstfer, Extension of G-quadruplex DNA by ciliate telomerase, The EMBO journal, 25 (2006) 1148-1159.

[3] S. Ceru, P. Sket, I. Prislan, J. Lah, J. Plavec, A new pathway of DNA G-quadruplex formation, Angewandte Chemie (International ed. in English), 53 (2014) 4881-4884.

[4] S. Artusi, M. Nadai, R. Perrone, M.A. Biasolo, G. Palu, L. Flamand, A. Calistri, S.N. Richter, The Herpes Simplex Virus-1 genome contains multiple clusters of repeated G-quadruplex: Implications for the antiviral activity of a G-quadruplex ligand, Antiviral research, 118 (2015) 123-131.

[5] J. Dai, T.S. Dexheimer, D. Chen, M. Carver, A. Ambrus, R.A. Jones, D. Yang, An intramolecular G-quadruplex structure with mixed parallel/antiparallel G-strands formed in the human BCL-2 promoter region in solution, Journal of the American Chemical Society, 128 (2006) 1096-1098.

[6] U. Sinha-Datta, S. Khan, D. Wadgaonkar, Label-free interaction analysis as a tool to demonstrate biosimilarity of therapeutic monoclonal antibodies, Biosimilars, 5 (2015) 83-91.

[7] X. Zhang, M. Oglesbee, Use of surface plasmon resonance for the measurement of low affinity binding interactions between HSP72 and measles virus nucleocapsid protein, Biological procedures online, 5 (2003) 170-181.
